# Patanjali and neuroscientific research on meditation

**DOI:** 10.3389/fpsyg.2015.00915

**Published:** 2015-07-03

**Authors:** Klaus B. Bærentsen

**Affiliations:** Department of Psychology and Behavioral Sciences, University of AarhusAarhus, Denmark

**Keywords:** Patanjali, meditation, brain processes, consciousness, resting state, contrastive method in fMRI, functional brain networks, dynamical systems

The definition of meditation (or yoga) by Patanjali as “restriction (or stilling) of the fluctuations of the mind” (cf, Woods, 1927/2003, p. xxx, 8) may be an appropriate starting point for research on meditation using fMRI. An operational definition of the neural substrate of meditation which is adequate to Patanjalis definition, may be developed on the basis of a non-reductionistic understanding of the neural underpinnings of the mind in terms of dynamical functional brain systems.

Awasthi (2013) argue that Patanjali belongs to a dualistic philosophical tradition (Sankhya), in which meditation is a spiritual phenomenon, inaccessible to objective research. But in the Sankhya philosophy, an intimate relationship is assumed to exist between the spiritual, including meditation, and the material concomitants of states of mind (Rao, 2011). Further, Patanjali suggested that a number of persistently motivated goal-directed sensory-motor activities, and socially oriented moral and mental exercises, must be accomplished in order to reach meditation (Woods, 1927/2003, p. xxx–xxxi, 34ff). Since neuroscience considers objectively measurable phenomena only, it may thus be impossible to investigate meditation as such, but the material concomitants of the mind during meditation and preceding exercises may be as accessible to objective research as during other states of mind. While the social and moral issues calls for other methods, some of the mental and concomitant sensory-motor exercises are accessible for objective neuroscientific investigation.

Awasthi (2013) and Rao (2011) express the need for a clear and commonly accepted definition of meditation to get beyond the present situation of mixed, and to some extent contradictory results. Increased attention to the practical details of the investigated specific forms of meditation, is necessary. They suggest an explicit consideration of what is meant by “meditation,” and of the corporeal operational details of the investigated activity, in order not to confuse various kinds and levels of meditation with each other. This is clearly relevant, and merits serious attention, but a thorough discussion of this topic is not possible here.

Another methodological problem also needs careful consideration. Most current research into meditation using PET, SPECT, or fMRI uses the contrastive method. The brain is scanned during various different states (at least two), one of which is the target state (meditation), and the other(s) some different state(s) of mind. The result is reached by subtracting the contrast state from the target state (cf, Raichle, 1998), and usually reported as (networks of) brain areas displaying increased or decreased activity levels during meditation. Although the results are usually described as information about the target state (meditation), they actually inform about relative differences in local brain activities related to changes of mind between various cognitive activities, including “mind wandering,” into the target state, attempted meditation (e.g., Bærentsen et al., 2010; Hasenkamp et al., 2012).

Simple arithmetic tells us that the result of this subtraction depends on both the subtrahend and the minuend, and the result is informative about the difference, not about the target state as such. Although some consensus has been reached, results may depart substantially from each other (cf, Cahn and Polich, 2006; Neumann and Frasch, 2006; Ivanovski and Malhi, 2007; Ospina et al., 2007; Tang et al., 2012). To some degree reflecting the confusion as to precisely what kind of meditative activity is investigated, and the contrasts that are used (Rao, 2011; Awasthi, 2013).

But maps of local activations and deactivations during “meditation” obtained with contrastive fMRI brain imaging only inform us of about approximately 1–5 % local variations in the level of neural activity (Raichle and Gusnard, 2005; Raichle and Mintun, 2006; Raichle, 2009). These variations are relevant, but contrary to what is often implicitly believed, the contrastive analysis provides us with *no knowledge* about the (equally intense) concurrent activity in the rest of the brain, apart from the fact, that there is no statistically significant difference in the level of neural activity between the contrasted states. But this is *not* information, that activity in the rest of the brain is *irrelevant* to the investigated states of mind, nor about what possible relation this ongoing activity may have to the investigated state of mind. Attempts to go beyond the concepts of activation and deactivation of local areas are represented by investigations of functional connectivity (e.g., Farb et al., 2007; Brewer et al., 2011; Hasenkamp and Barsalou, 2012; Pagnoni, 2012; Taylor et al., 2013; Josipovic, 2014), but although interesting, a thorough consideration of these efforts is impossible here.

Patanjalis definition consider meditation and its obstacles, i.e., flutuations of the mind. According to Patanjali, five types of fluctuations of mind should be overcome in order to attain concentration (meditation): (1) veridical cognition on the basis of perception, logical reasoning, and verbal communication, (2) illusory imagination, (3) linguistic conceptualizations, (4) sleep, and (5) memory (Woods, 1927/2003; Rao, 2011, p. xxx, 17ff). These fluctuations corresponds to, roughly speaking, the full range of mental states which the mind may pass through during normal life activity as they are described in modern psychology.

All of these different “states of mind” in activity are more or less goal-directed (e.g. internally vs. externally defined) and characterized by continuously changing constellations of motivations, goal-related cognition-emotion, and operational regulatory mental and sensory-motor processes (Leont'ev, 1974; Leontev, 1978). They are realized by task-related functional brain systems integrating functionally differentiated and anatomically distributed areas of the whole brain, not by patches of local “activated” areas (Brodmann, 1909/1985; Vygotsky, 1934/1997; Anokhin, 1969, 1974; Luria, 1973; Edelman and Tononi, 2000; Freeman, 2000; Raichle and Gusnard, 2005). The evolution of the functional brain systems is reflected in changing patterns of local activations and deactivations as revealed by fMRI. They correspond to the establishment and dissolution of functional synchronization of neural activity in distributed local populations of synapses (Magri et al., 2012), reflecting changes in intentional focus, emotional coloring, cognitive strategies, and processing routines etc. One example is the change from mind wandering (whatever its specific content) into concentration on a chosen meditative focus (e.g., Guo and Pagnoni, 2008; Bærentsen, 2011; Hasenkamp et al., 2012).

Even if it would be possible to control the exact nature of the momentary meditative focus and the qualitative contents of the contrasting “mind wandering” episode, it is however unlikely, that any two instances would be reflected in identical patterns of local activations and deactivations (cf, McGonigle et al., 2000). Meditation as such is probably not reflected in a specific momentary pattern of “activations” and “deactivations,” as these are currently understood. But meditation may correspond to a specific kind of global dynamic of changing systems of coactivated brain areas, and this dynamic may be different from those that corresponds to the five mentioned “fluctuations” during mundane states of consciousness.

Patanjali may view meditation as a spiritual phenomenon, but it is not stated in the sutras that the material concomitants of mind are irrelevant to the mentioned fluctuations, nor that they cease to exist if the spirit eventually attains liberation. Although different schools of meditation use specific techniques and procedures (Focused Attention, Open Monitoring, Nondual Awarenes etc., cf, Josipovic, 2014), the ultimate aim always includes some form of “restriction (or stilling) of the fluctuations of the mind.” Assuming that meditation entails brain activity that differ from common resting or various goal-directed mental activities including various techniques of meditation exercise (Guo and Pagnoni, 2008; Bærentsen, 2011; Hasenkamp et al., 2012), neuroscience research may try to unveil the dynamic characteristics of the material concomitants of mind during various kinds and levels of meditation (cf, Rao, 2011), with or without the use of the contrastive method (cf, Beckmann et al., 2005; Guo and Pagnoni, 2008; Smith et al., 2009).

Meditation is a whole-brain (body) activity and needs characterization in terms of whole brain dynamics. The research strategy might follow the non-reductionist proposal of Edelman (2003) and Edelman and Tononi (2000 p. 18f) and concentrate on the brain processes, not just the brain areas, that support consciousness, and examine what kind of neural interactions may explain the fundamental properties of consciousness such as phenomenological unity, differentiation, variability and informativeness, that may reveal characteristics conforming to the phenomenological descriptions of the target states (e.g., rest vs. various forms and states of meditation).

In terms of dynamical systems, states of mind may be considered and understood as temporary stable patterns of activity, i.e., as trajectories in bounded regions of a state space created by the evolution of the global patterns of activity in the brain (cf, Edelman and Tononi, 2000; Freeman, 2000; Haken and Schiepek, 2010). Each state may be of varying temporal duration, and more or less stable according to the characteristics of the attractors and control parameters (e.g., arousal, motivation, concentration, skill). In stable states, it will remain in a bounded area of state space. During state transitions it may reveal characteristic instabilities etc. States like mind wandering and meditation may pertain to various phases and show characteristic patterns of stability, fluctuations, instabilities, transitions between states, etc.

The descriptions given by Patanjali provides references to which types of states could be compared. These conventional states of mind define trajectories in the brain's state space—and may well be described as “fluctuations” therein, more or less intense according to the level of wakefulness, the amount of drive, levels of motivation or intensity of emotional content etc. They may be more or less coherent, integrated, and stable according to the level of concentration on the intended goal or direction, and they may be more or less qualitatively differentiated and encompassing according to the level of operational sensory-motor coordination to the situational conditions, and their emotional color.

In everyday activities, conscious attention may be variously focused on a particular intentional goal to achieve, a problem to solve, the aesthetic or emotional qualities of a situation, etc. During resting when no explicit goal is aimed at, attention is moving from one topic to another and focus on varying qualities and aspects of imagined situations according to drives, previous experiences, distractions and random influences (cf, Smallwood and Schooler, 2006; Schooler et al., 2011) as reflected in the fluctuations in ongoing brain activity (Raichle, 2011).

During the practice of meditation one may suddenly realize that the mind has been caught by thoughts about some issue and that awareness of the present situation and the goal has faded. In such cases the mind may be refocused on the situation and the intended action when awareness of the distraction emerges in consciousness (Hasenkamp et al., 2012). The contents of perception or thoughts, and the focus of conscious attention or mind, may not be identical, and during the transition from one issue to the other, it is not residing in neither of the contents (Schooler et al., 2011). Before and after the transition however, the conscious mind usually resides in (i.e., identifies with) the content, i.e., the fluctuations, and these fluctuations are reflected in the measurable activity of the brain (Hasson et al., 2004).

It may be expected, that the fluctuations are large, when the mind is caught emotionally and engages in cognitive imagination with a rich and varied qualitative content. When meditation is achieved, the mind does—according to Patanjali—not identify with these “fluctuations,” and the concomitant fluctuations of brain activity are possibly minimal (controlled, stilled, suppressed), as reflected in the Buddhist expression “The flame will only burn steadily when we can calm the air around it…when we have stilled the turbulence of our thoughts and emotions” (Rinpoche, 1992, p. 64). But if “stillness” is conceived of as “being balanced” in relation to fluctuations and disturbances, it may actually be expressed in compensatory activities to counterbalance these influences. “once we have found a stability in our meditation, noises and disturbances of every kind will have far less impact” (Rinpoche, 1992, ibid.).

Stillness may be understood as controlled variability counterbalancing “fluctuations” of the mind (Hasenkamp et al., 2012), which is reminiscent of the discussion about whether “stilling the fluctuations” or “relaxation” entails lowering arousal, which seems not to be the case (Bærentsen et al., 2010; Amihai and Kozhevnikov, 2014).

How the different states of mind may be reflected in the different measurable fluctuations of global brain processes is a question to be asked.

## Conflict of interest statement

The author declares that the research was conducted in the absence of any commercial or financial relationships that could be construed as a potential conflict of interest.
